# Phenotype and genotype in hereditary chronic intestinal pseudo-obstruction with small intestine involvement

**DOI:** 10.3389/fmed.2025.1632816

**Published:** 2025-08-21

**Authors:** Yang Chen, Xueyan Chen, Chengzhu Ou, Yiliang Chen, Ji Li, Jingnan Li, Yaping Liu, Xiaoqing Li

**Affiliations:** ^1^Department of Gastroenterology, Peking Union Medical College Hospital, Chinese Academy of Medical Sciences and Peking Union Medical College, Beijing, China; ^2^Qingdao Medical College, Qingdao University, Qingdao, China; ^3^Center for Rare Diseases, Peking Union Medical College Hospital, Chinese Academy of Medical Sciences and Peking Union Medical College, Beijing, China

**Keywords:** hereditary chronic intestinal pseudo-obstruction, phenotype, genotype, small intestine involvement, gene mutation

## Abstract

Chronic intestinal pseudo-obstruction (CIPO) is a rare and severe intestinal motility disorder with poor long-term prognosis and high mortality rate, especially when the small intestine is involved. Due to the non-specificity of clinical symptoms, CIPO has long faced diagnostic challenges. With the advancements of sequencing technology, many hereditary CIPOs have been identified. Establishing the relationship between genotype and phenotype of hereditary CIPO to make diagnosis early has become a focal point for clinicians. This article reviewed hereditary CIPO with small intestine involvement reported in the past 25 years, collecting patients’ phenotypic and genetic information, and categorizing them into several groups for comparative analysis based on the involved intestinal segments and pathological features. A total of 75 cases were included. We found that the CIPO group with both small and large intestine involvement (SLI) had a higher proportion of bloating and constipation, while the CIPO group with isolated small intestine involvement (ISI) had a higher proportion of diarrhea and was more likely to be associated with mitochondrial disorders. Hereditary CIPO patients associated with mitochondrial disorders exhibited a later age of onset, higher prevalence of malnutrition, and more prominent multi-system involvement. Other myogenic CIPO patients, in which *ACTG2* was the most frequently mutated gene, showed more frequent SLI and a high incidence of malrotation. This article preliminarily explores the correlation between genotype and phenotype in hereditary CIPO, focusing specifically on patients with small intestine involvement, aiming to provide valuable clues for the early identification and diagnosis of hereditary CIPO with small intestine involvement.

## 1 Introduction

Chronic intestinal pseudo-obstruction (CIPO) is a rare gastrointestinal disorder characterized by impaired intestinal motility. This uncommon condition is marked by recurrent or persistent symptoms resembling intestinal obstruction, including abdominal pain, bloating, vomiting, and constipation, without any evidence of mechanical obstruction (1). According to the nationwide surveys in Japan, the prevalence of CIPO was approximately 1.00 case per 100,000 individuals in males and 0.80 case per 100,000 individuals in females (2). Additionally, the pediatric prevalence was documented as 0.37 cases per 100,000 (3). The incidence was reported as 0.21 and 0.24 cases per 100,000 individuals in males and females, respectively (2).

Chronic intestinal pseudo-obstruction can be classified into primary and secondary categories based on the etiology. Primary CIPO is caused by hereditary neuromuscular diseases with genetic origin. Secondary CIPO results from definite secondary factors, such as systemic diseases, endocrinological disorders, tumors, drugs, or infections. Idiopathic CIPO is with unknown etiology (4, 5). With the advancement of genetics, some characteristic germline mutations, such as *ACTG2*, *RET*, and *TYMP* gene mutations are identified in primary CIPO, which are classified as hereditary CIPO. These genes encode actin, regulate enteric nervous system development, or are involved in mitochondrial nucleotide metabolism. Their mutations disrupt the normal function of the muscles, intestinal nerves or interstitial cells of Cajal (ICC), thereby leading to different pathological changes and intestinal dysmotility (6). In recent years, high-throughput sequencing has enabled more hereditary CIPO patients to receive molecular diagnoses and has provided additional material for genetic research. Besides, CIPO could involve small intestine, large intestine, or both. Patients with small intestine involvement often exhibit more severe manifestations and poorer surgical outcomes, highlighting the need for extra attention and becoming the focal point of this review (7).

Previous studies indicate that hereditary CIPO has a poor long-term prognosis and a high mortality rate (8, 9). The mortality rate is approximately 10% in adults and can be as high as 25% in pediatric populations (10, 11). Moreover, due to the low specificity and high heterogeneity of clinical symptoms, CIPO also presents a significant diagnostic challenge (9). Many patients with ambiguous or misdiagnosed conditions undergo abdominal surgery, which may further distress their compromised bodies (1). Empirical evidence has shown that surgical intervention, such as laparotomy and intestinal resection, not only fails to improve the condition but may also exacerbate the patient’s symptoms (5). Severe intestinal motility disorders can ultimately progress to intestinal failure, which may result in patient mortality. Therefore, early identification of hereditary CIPO patients, along with appropriate interventions and avoiding unnecessary invasive procedures, can significantly improve prognosis and enhance survival rates (12, 13).

Although there is the presence of a relatively clear etiology, it is still challenging to identify hereditary CIPO in early stage, especially for patients with small intestine involvement. For clinicians, the specific clinical scenarios that necessitate consideration of CIPO with different intestinal segments involvement, as well as the differential manifestations that might indicate CIPO caused by different gene mutations, which lead to different pathological changes, remain inadequately defined. To address this gap, we conducted a comprehensive review of case reports on hereditary CIPO published in the past 25 years. Our aim was to explore the common genetic scenarios in CIPO patients with small intestine involvement, and to investigate the potential correlation between clinical phenotypes and genetic backgrounds.

## 2 Methods

### 2.1 Search strategies

In the PubMed database, a search strategy combining MeSH terms with free text was employed to construct a search query, which yielded 750 English articles spanning 25 years from January 1, 2000, to January 1, 2025 (detailed search query in the Supplementary material). The following search terms were used: “Intestinal Pseudo-Obstruction,” “CIPO,” “small intestine,” “small bowel,” “Duoden*,” “ileo*,” “jejun*,” “aganglionosis,” “hypoganglionosis,” “Visceral Myopath*,” “Enteric Neuropath*,” “gene*,” “heredit*,” “mutation*,” “variant*,” etc. Out of the 731 articles accessible in full text, 591 were excluded based on title and abstract scan. A detailed reading of the remaining 140 articles identified 51 articles suitable for extracting case information on hereditary CIPO with small intestine involvement. Two independent researchers conducted the process and the consistency was checked by a third researcher.

### 2.2 Criteria for case selection

Cases that fulfilled all the following criteria were included: (1) confirmed diagnosis of primary CIPO characterized by typical symptoms of obstruction such as abdominal pain and distension, images showing bowel dilation without evidence of mechanical obstruction (7, 14); (2) involvement of small intestine; (3) availability of hereditary information (with identified mutated genes).

Cases were excluded if any of the following applied: (1) acute intestinal pseudo-obstruction or secondary CIPO; (2) lack of imaging evidence for bowel dilation; (3) without involvement of the small intestine or unclear involvement site of the intestinal tract; (4) absence of hereditary information; (5) insufficiently detailed case reports that prevented the extraction of required information.

**Figure 1:**
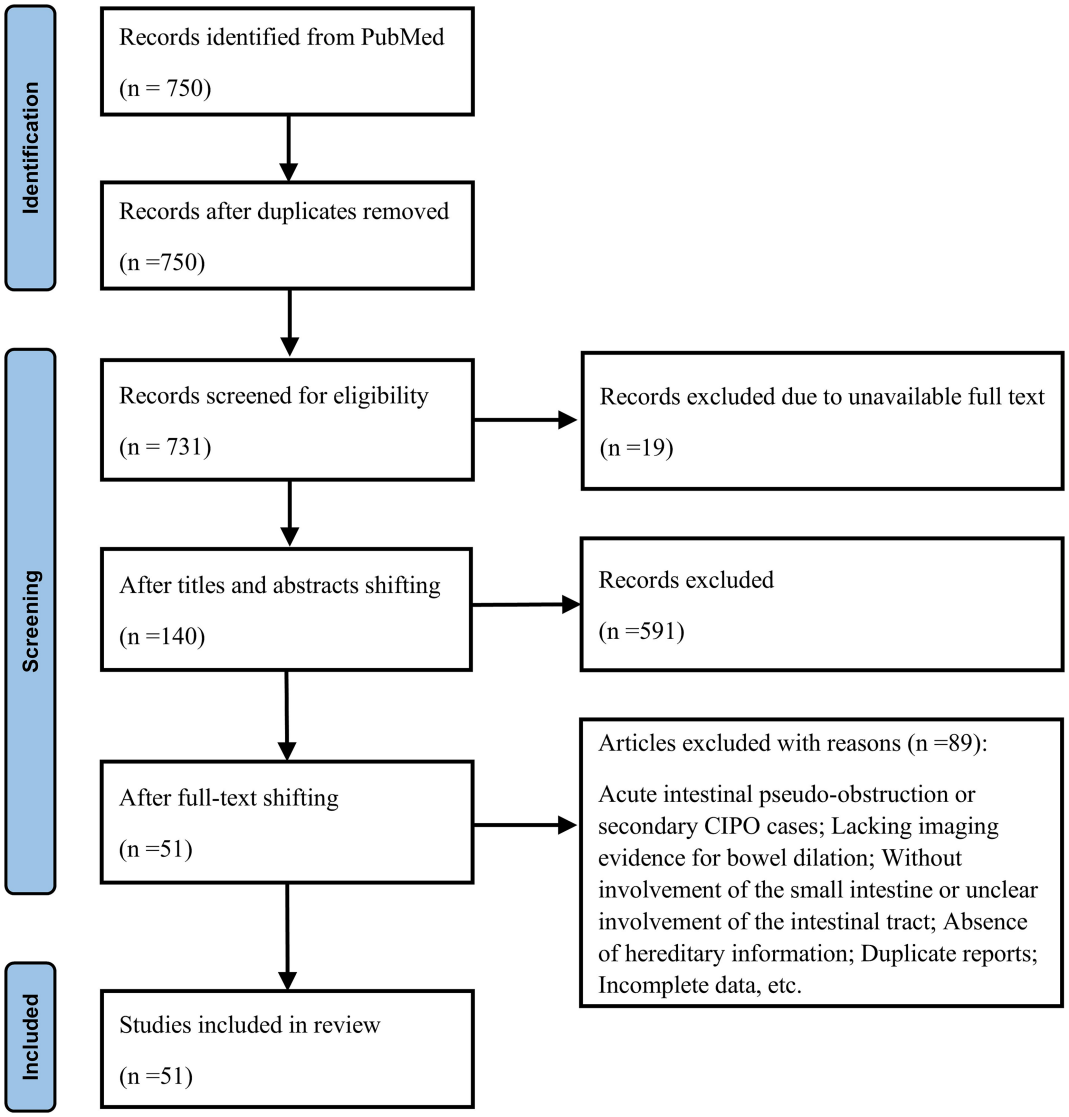
Flowchart summarizing the process of record screening.

Finally, 75 cases satisfying the inclusion criteria were extracted from 51 articles (Figure 1).

### 2.3 Data extraction

We documented the hereditary CIPO patients’ demographic characteristics (such as gender, age of onset, age at diagnosis, family history, etc.), clinical features (including CIPO-related gastrointestinal symptoms, other intestinal symptoms, nutrition condition, phenotypic syndromes, etc.), interventions (the proportion receiving total parenteral nutrition support and history of abdominal surgery), genetic information (mutated genes, variations, inheritance patterns, etc.).

For hereditary CIPO patients with extra-gastrointestinal involvement, the age of onset was defined as the age when gastrointestinal symptoms first appeared, while the age at diagnosis was when CIPO was confirmed. Patients with onset and diagnosis before birth were assigned an age of 0 for data analysis. Malnutrition was recorded in patients experiencing significant weight loss, decreased BMI, anemia, and hypoproteinemia since disease onset.

Patients were categorized into two groups according to involved intestinal segments: CIPO with isolated small intestine involvement (CIPO-ISI) and CIPO with both small and large intestine involvement (CIPO-SLI), based on intestinal dilation sites identified via X-ray, CT, MRI, or endoscopy. According to pathology, patients were classified into five groups: neurogenic CIPO, myogenic CIPO, CIPO associated with mitochondrial disorders, interstitial lesion CIPO, and mixed-type CIPO (6, 15). Traditionally, CIPO associated with mitochondrial disorders was included in the myogenic group. However, it has unique mutated genes that distinguish it from other myogenic types, such as *TYMP* and *POLG*, and is rooted in cellular energy failure rather than destruction of muscle fibers or neural structures, which leads to unique clinical manifestations, such as a late age of onset. The etiology of energy metabolism disorders also determines that it can be diagnosed by detecting energy metabolism markers and unique treatment strategies for metabolic support can be explored, which is much different from other myogenic types. These are of great significance for understanding the occurrence and development of diseases and exploring new treatments. Therefore, myogenic CIPO (excluding mitochondrial disorders) and CIPO associated with mitochondrial disorders were analyzed separately in this review. Data analysis was not performed for interstitial lesion CIPO and mixed-type CIPO due to the extremely small and unrepresentative number of cases collected.

### 2.4 Statistical analysis

**Figure 2:**
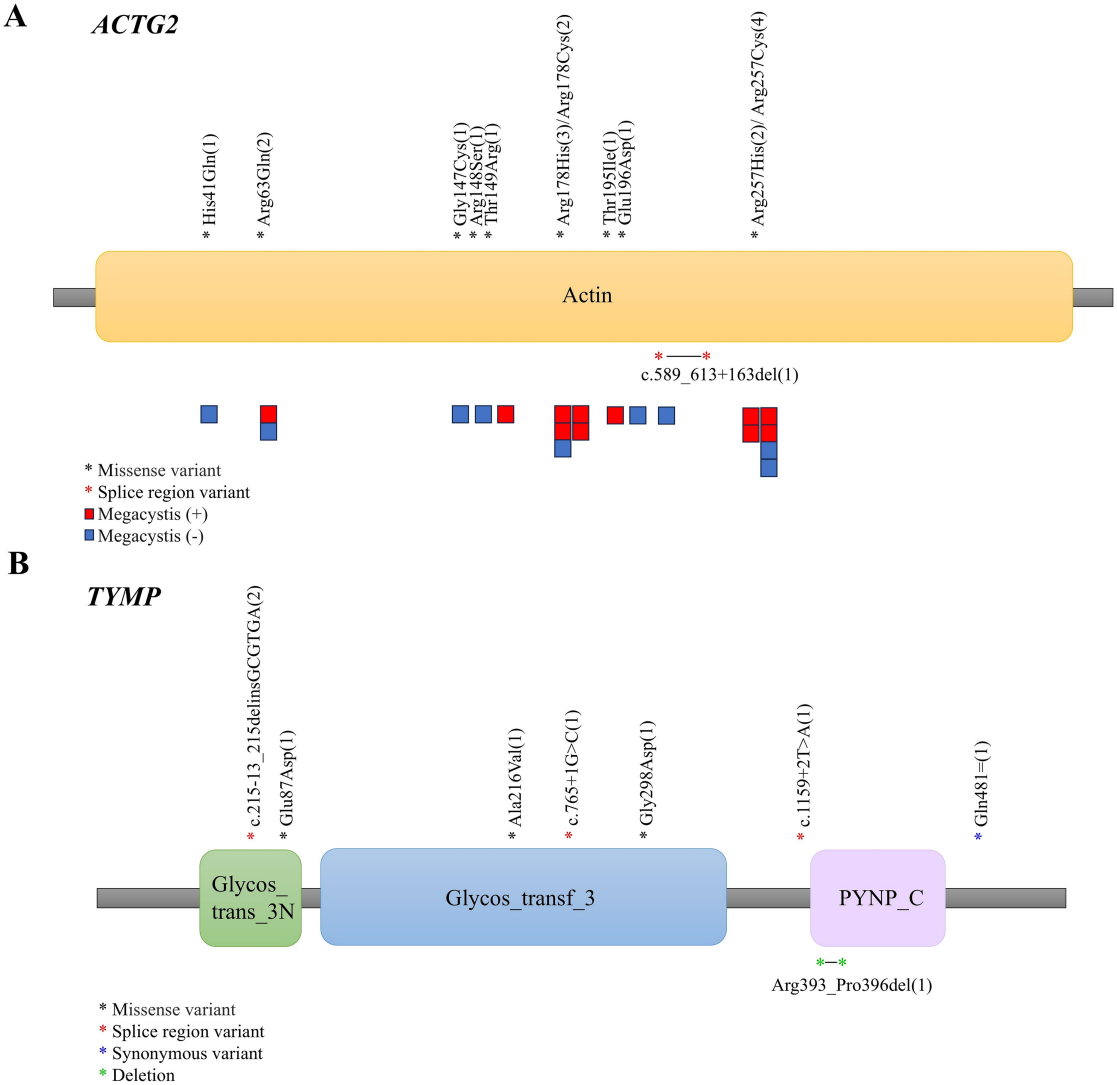
Schematic diagram showing the location of variant sites in the *ACTG2* **(A)** and *TYMP* **(B)** domains.

The Kolmogorov–Smirnov test was employed to assess whether the measurement data followed a normal distribution. Continuous variables were shown as mean ± standard deviation (SD) for those fitting a normal distribution and median (interquartile range [IQR]) for those not. Student’s t-test and Mann–Whitney U-test were used for comparison analysis, respectively. Categorical variables were reported as numbers and percentages and compared by Fisher’s exact test between the two groups. A *p*-value less than 0.05 was considered statistically significant. The R epitools package was used to calculate RR and 95% confidence intervals (with SLI as an exposure group). Statistical analyses were performed using the R language software. In Figure 2, squares represented patients, with colors indicating megacystis status. Each patient’s condition was listed below their variation type to visualize the relationship between the two.

## 3 Results

### 3.1 General characteristics of hereditary CIPO patients

Among 75 patients, there was a slight male predominance (56%, 42/75). The median ages of onset and diagnosis were 0.05 (0.00, 15.00) years and 3.00 (0.17, 30.00) years. Interestingly, 4 patients were detected with bowel dilation before birth.

The common gastrointestinal symptoms included bloating (65.67%), nausea or vomiting (62.69%), abdominal pain (49.25%), constipation (47.76%), and diarrhea (35.82%). 40.00% of patients had malnutrition, manifesting as weight loss, decreased BMI, anemia, and hypoproteinemia. Finally, 29 (38.67%) patients received TPN support. Among all patients, more than half (62.67%, 47/75) have undergone abdominal surgery, including procedures such as laparotomy, intestinal resection, stoma creation, or intestinal transplantation (16) (Table 1).

**Table 1:** Demographic and clinical information of patients with hereditary CIPO.

Characteristics		Total (*n* = 75)	Site of involvement		RR (95%CI)	*p*-value	Pathological types			*P*-value
			Isolated small intestine involvement (*n* = 34)	Both the small and large intestines involvement (*n* = 41)			myogenic CIPO (*n* = 29)	CIPO associated with mitochondrial disorders (n = 17)	neurogenic CIPO (*n* = 25)	
Demographic information										
Sex (male/female)		42/33	24/10	18/23		0.037	16/13	11/6	12/13	0.565
Age of onset	Unknown (n)	7	6	1			1	2	2	
	Onset before birth (n)	5	1	4			5	0	0	
	Median age (year)	0.05 (0.00,15.00)	11.00 (0.05, 21.25)	0.00 (0.00, 1.44)		<0.001	0.00^a^ (0.00, 0.31)	15.00^b^ (4.50, 27.00)	0.01 (0.00, 14.50)	<0.001
Age at diagnosis	Unknown (n)	30	14	16			8	6	13	
	Prenatal diagnosis (n)	1	0	1			0	0	1	
	Median age (year)	3.00 (0.17, 30.00)	21.00 (0.58, 42.25)	1.25 (0.17, 15.00)		0.123	0.58^a^ (0.03, 2.00)	23.00 (18.50, 33.50)	24.00 (0.58, 46.50)	0.007
Interval from onset to diagnosis	Unknown (n)	32	16	16			9	7	13	
	Median interval (year)	0.96 (0.02, 9.00)	8.50 (0.19, 22.38)	0.58 (0.00, 3.00)		0.039	0.50 (0.00, 1.50)	3.00 (0.01, 8.75)	7.00 (0.40, 22.75)	0.147
Family history	*N* (percent)	37 (57.81%)	24 (82.76%)	13 (37.14%)		0.002	10 (40.00%)^a^	10 (90.91%)	14 (58.33%)	0.015
	Unknown (n)	11	5	6			4	6	1	
Clinical characteristics										
CIPO-related gastrointestinal symptoms										
Abdominal pain	*N* (percent)	33 (49.25%)	23 (74.19%)	10 (27.78%)	0.36 (0.19–0.67)	<0.001	5 (23.81%)^a^	13 (76.47%)	12 (48.00%)	0.005
Bloating	*N* (percent)	44 (65.67%)	15 (48.39%)	29 (80.56%)	2.65 (1.26–5.60)	0.012	19 (90.48%)	9 (52.94%)	12 (48.00%)^c^	0.007
Nausea and vomiting	*N* (percent)	42 (62.69%)	16 (51.61%)	26 (72.22%)	1.74 (0.92–3.30)	0.137	10 (47.62%)	13 (76.47%)	18 (72.00%)	0.116
Diarrhea	*N* (percent)	24 (35.82%)	19 (61.29%)	5 (13.89%)	0.45 (0.28–0.71)	<0.001	4 (19.05%)	7 (41.18%)	11 (44.00%)	0.171
Constipation	*N* (percent)	32 (47.76%)	9 (29.03%)	23 (63.89%)	1.97 (1.20–3.21)	0.009	10 (47.62%)	6 (35.29%)	15 (60.00%)	0.286
	Unknown (n)	8	3	5			8	0	0	
Other intestinal symptoms										
Malrotation	*N* (percent)	13 (17.33%)	5 (14.71%)	8 (19.51%)	1.06 (0.86–1.30)	0.810	10 (34.48%)^a^	0 (0.00%)	3 (12.00%)	0.007
Malnutrition	*N* (percent)	30 (40.00%)	16 (47.06%)	14 (34.15%)	0.80 (0.55–1.18)	0.368	12 (41.38%)	14 (82.35%)^b^	3 (12.00%)	<0.001
Total parenteral nutrition	*N* (percent)	29 (38.67%)	10 (29.41%)	19 (46.34%)	1.32 (0.92–1.88)	0.207	16 (55.17%)	6 (35.29%)	5 (20.00%)	0.028
Abdominal surgery	*N* (percent)	47 (62.67%)	19 (55.88%)	28 (68.29%)	1.39 (0.77–2.50)	0.386	23 (79.31%)^a^	6 (35.29%)	15 (60.00%)	0.012

^a^When myogenic CIPO and CIPO associated with mitochondrial disorders are compared, *p* < 0.05/3.

^b^When CIPO associated with mitochondrial disorders and neurogenic CIPO are compared, *p* < 0.05/3.

^c^When neurogenic CIPO and myogenic CIPO are compared, *p* < 0.05/3.

In terms of genetic information, 75 hereditary CIPO cases included 29 myogenic (38.67%), 17 mitochondrial disorder-associated (22.67%), 25 neurogenic (33.33%), 1 interstitial (1.33%), and 3 mixed type (3.99%). There were 50 kinds of variations recorded in Table 2, with pathogenic and likely pathogenic variations accounting for 68.00% (34/50). Among all the mutated genes, *ACTG2* was the most common (28.00%, 21/75), involving 12 kinds of variations (Figure 2A). And 8 of these mutations were classified as pathogenic by the Clinvar submitters. The condition of megacystis in patients was also shown in the figure, but no significant association was found between variations and megacystis. *TYMP* ranked second in frequency (13.33%, 10/75), involving 10 variations (Figure 2B), 6 of which were pathogenic or likely pathogenic.

**Table 2:** Genetic information of hereditary CIPO patients.

Pathological types	Gene	Map of position	Variation	Interpretation in Clinvar*	Number of cases	Zygosity	Ref.	Genetic mode	isolated small intestine involvement (*n* = 34)	both the small and large intestines involvement (*n* = 41)
Myogenic CIPO	ACTG2	2p13.1	NM_001615.4(ACTG2):c.123C > A (p. His41Gln)	VUS (1)	1	Heterozygous	(24)	AD/AR	2	19
			NM_001615.4(ACTG2):c.188G > A (p. Arg63Gln)	PAT (1)	2	Heterozygous	(34)			
			NM_001615.4(ACTG2):c.442C > A (p. Arg148Ser)	PAT (1)	1	–	(35)			
			NM_001615.4(ACTG2):c.446C > G (p. Thr149Arg)	VUS (1)	1	Heterozygous	(24)			
			NM_001615.4(ACTG2):c.532C > T (p. Arg178Cys)	PAT (6)	2	Heterozygous	(18, 36)			
			NM_001615.4(ACTG2):c.533G > A (p. Arg178His)	PAT (3)	3	Heterozygous	(34, 37)			
			NM_001615.4(ACTG2):c.584C > T (p. Thr195Ile)	PAT (1)/VUS (1)	1	Heterozygous	(38)			
			NM_001615.4(ACTG2):c.588G > C (p. Glu196Asp)	PAT (1)	1	–	(39)			
			NM_001615.3(ACTG2):c.589_613 + 163del	LP (1)	1	Homozygous	(19)			
			NM_001615.4(ACTG2):c.769C > T (p. Arg257Cys)	PAT (15)	4	Heterozygous	(24, 37, 40)			
			NM_001615.4(ACTG2):c.770G > A (p. Arg257His)	PAT (3)	2	Heterozygous	(37)			
			NM_001615.4(ACTG2):c.439G > T (p. Gly147Cys)	LP	1	Heterozygous	(41)			
	FLNA	Xq28	NM_001110556.2(FLNA):c.18_19del (p. Arg7GlyfsTer98)	PAT (2)	1	Hemizygous	(42)	XLR	4	0
			NM_001110556.2(FLNA):c.7021C > T (p. Gln2341Ter)	PAT (1)	1	Hemizygous	(43)			
	MYH11	16p13.11	NM_001040113.2(MYH11):c.5819del (p. Pro1940HisfsTer91)	LP (4)/VUS (2)	1	Heterozygous	(4)	AD	1	0
	LMOD1	1q32.1	NM_012134.3(LMOD1):c.1106C > T (p. Thr369Met)	PAT (1)	1	Compound heterozygous	(44)	AR	1	0
			NM_012134.3(LMOD1):c.1262G > A (p. Arg421His)	PAT (1)						
	EVC2	4p16	NM_147127.5(EVC2):c.2653C > T (p. Arg885Ter)	PAT (2)	1	Compound heterozygous	(45)	AR	0	1
			NM_147127.5(EVC2):c.1814C > A (p. Ser605Ter)	PAT						
	MYL9	20q11.23	NM_006097.5(MYL9):c.184 + 2_184 + 10del	VUS (1)	1	Compound heterozygous	(46)	AR	0	1
			NM_006097.5(MYL9):c.347-21_*594del	–						
Neurogenic CIPO	RET	10q11.2	NM_020975.6(RET):c.341G > A (p. Arg114His)	BEN (8)/LB (2)	1	-	(20)	AD	1	4
			NM_020975.6(RET):c.1852 T > C (p. Cys618Arg)	PAT (10)	1	Heterozygous	(21)			
			NM_020975.6(RET):c.1523-1G > C	–	2	Heterozygous	(13)			
	PHOX2B	4p13	NM_003924.4(PHOX2B):c.693_700del (p. Pro232ArgfsTer125)	–	1	Heterozygous	(47)	AD	0	1
	FLNA	Xq28	–	–	1	–	(17)	XLR	1	0
	KIF26A	11q13.3	NM_015656.2(KIF26A):c.4085dup (p. Ala1363GlyfsTer47)	PAT (1)	1	Homozygous	(48)	–	0	1
	9p21.3 dup	9p21.3	g.20900000_22100000dup (GCF_000001405.25_GRCh37.p13; 2017 04–19)	–	6	–	(49)	AD	5	1
	EDNRB	13q22.3	NM_001122659.3(EDNRB):(p. Ser196Asn)	–	1	Homozygous	(50)	AR	0	1
	TCF4	18q21.2	NM_001083962.2(TCF4):c.1738C > T (p. Arg580Trp)	PAT (1)	1	Homozygous	(51)	–	0	1
	RAD21	8q24.11	NM_006265.3(RAD21):c.1864G > A (p. Ala622Thr)	PAT (1)	3	Homozygous	(52)	AR	3	0
	CLMP	11q24.1	g.122953792-122955421del (HGL9)	–	1	Homozygous	(53)	AR	0	1
	TCOF1	5q32	NM_001371623.1(TCOF1):c.4369_4372del (p. Glu1457ArgfsTer118)	PAT (1)/LP (1)	1	Heterozygous	(54)	AD	1	0
	TTC7A	17q25.3	NM_020458.4(TTC7A):c.974G > A (p. Arg325Gln)	VUS (4)/LB (1)	1	Homozygous	(55)	–	1	0
	NXPH4	12q13.3	g.55822753_55917767 (hg18)	–	1	–	(56)	–	0	1
	DDX3X	Xp11.4	NM_001356.5(DDX3X):c.1574A > G (p. Tyr525Cys)	LP (1)	1	Heterozygous	(57)	XLD	0	1
	TFAP2B	6q22.1	NM_003221.4(TFAP2B):c.602-5_606del	PAT (1)	1	Heterozygous	(58)	AD	1	0
CIPO associated with mitochondrial disorders	MT-TL1 (A3243G)	m.3243	NC_012920.1:m.3243A > G	PAT (24)/LP (2)	3	-	(59–61)	AR	0	6
	TYMP	22q13.33	NM_001953.5(TYMP):c.215-13_215delinsGCGTGA	LP (1)	2	Compound heterozygous	(62)	AR	10	0
			NM_001953.5(TYMP):c.1159 + 2 T > A	PAT (3)						
			NM_001953.5(TYMP):c.261G > T (p. Glu87Asp)	PAT (1)	1	Compound heterozygous	(63)			
			g.49275958_49451008del (NCBI Build 36.1)	–						
			g.5044_5061dup (GenBank accession # M58602)	–	1	Homozygous	(64)			
			NM_001953.5(TYMP):c.647C > T (p. Ala216Val)	LP (2)	1	Homozygous	(65)			
			NM_001953.5(TYMP):c.765 + 1G > C	LP (1)	1	Homozygous	(66)			
			NM_001953.5(TYMP):c.893G > A (p. Gly298Asp)	LP (3)	1	Homozygous	(67)			
			NM_001953.5(TYMP):c.1176_1187del (p. Arg393_Pro396del)	–	1	Homozygous	(68)			
			NM_001953.5(TYMP):c.1443G > A (p. Gln481=)	VUS (3)/LB (3)	1	Homozygous	(69)			
	POLG	15q25.3	NM_002693.3(POLG):c.679C > T (p. Arg227Trp)	PAT (8)/LP (3)	1	Compound heterozygous	(70)	AR	0	1
			NM_002693.3(POLG):c.2542G > A (p. Gly848Ser)	PAT (29)						
interstitial lesion CIPO	L1CAM	Xq28	NM_001278116.2(L1CAM):c.2920G>T (p. Gly974Thr)	PAT (1)	1	-	(71)	XLR	0	1
mixed-type CIPO	8q23-q24	8q23-q24	–	–	3	–	(72)	AR	3	0

VUS, variant of uncertain significance; PAT, pathogenic; LP, likely pathogenic; BEN, benign; LB, likely benign; AD, autosomal dominant; AR, autosomal recessive; XLD, X-linked dominant; XLR, X-linked recessive. *Arabic numerals in the parentheses indicate the number of submitters.

### 3.2 Characteristics of hereditary CIPO patients with different involved sites

When patients were categorized into ISI and SLI groups, there was a male prevalence in the ISI group compared with the SLI group (70.59% vs. 43.90%). The median age of onset in the SLI group was 0.00 (0.00, 1.44) years, significantly earlier than that in the ISI group (11.00 years, Q1–Q3: 0.05, 21.25).

A comparison of these two groups revealed that the ISI group had a significantly higher proportion of patients experiencing abdominal pain (74.19% vs. 27.78%, *p* < 0.05) and diarrhea (61.29% vs. 13.89%, *p* < 0.05). Conversely, the SLI group had a higher probability of experiencing bloating (80.56% vs. 48.39%, *p* < 0.05) and constipation (63.89% vs. 29.03%, *p* < 0.05). Nausea and vomiting were common in both groups. Additionally, small intestinal bacterial overgrowth (SIBO) was detected in 8 cases in the ISI group. More than half of the patients in both groups had undergone abdominal surgery. In terms of genetics, a higher proportion of patients in the ISI group reported a positive family history (82.76% vs. 37.14%, *p* < 0.05). Among patients with ISI, *TYMP* mutations accounted for the highest proportion (29.41%, 10/34), whereas *ACTG2* mutations predominated in the SLI patients (46.34%, 19/41).

### 3.3 Characteristics of hereditary CIPO patients with different pathological types

When grouped according to pathological type, the proportion of myogenic, neurogenic, and mitochondrial disorder-associated CIPO were 38.67%, 33.33% and 22.67%, respectively. Patients with myogenic (0.00 year) and neurogenic (0.01 year) etiologies had a significantly earlier median age of onset, compared with patients with mitochondrial disorders (15.00 year). However, neurogenic CIPO had a relatively later diagnostic age with a median interval of 7.00 years from onset to diagnosis.

Patients with mitochondrial disorders experienced more abdominal pain (76.47%, 13/17) and showed a higher proportion of positive family history (90.91%, 10/11) than myogenic group, and had a higher incidence of malnutrition (82.35%, 14/17) compared to neurogenic group. Additionally, myogenic patients experienced more bloating (90.48%, 19/21) than neurogenic type, and showed a higher proportion of malrotation (34.48%, 10/29) compared to patients with mitochondrial disorders.

Among myogenic patients, those with SLI significantly outnumbered the ISI group (72.41% vs. 27.59%). Notably, in the myogenic CIPO patients with representative mutation gene *ACTG2*, SLI patients accounted for as high as 90.48% (19/21). According to the data, the most frequently mutated genes in myogenic CIPO were *ACTG2* (72.41%, 21/29) and *FLNA* (13.79%, 4/29), while in neurogenic CIPO, the prevalent mutations were *RET* (20%, 5/25) and 9p21.3 duplication (24%, 6/25). In mitochondrial lesion-related CIPO, *TYMP* (58.82%, 10/17) and *A3243G* (35.29%, 6/17) were more frequently observed. All 10 patients with *TYMP* mutations presented with ISI, while all 6 patients with *A3243G* mutations presented with SLI.

Additionally, among patients with mutations in the same gene, clinical and genetic characteristics can vary significantly due to differences in mutation types. For instance, *FLNA* mutations can lead to both myogenic CIPO and neurogenic CIPO (17). *ACTG2* mutations can manifest as either autosomal dominant or, in rare cases, autosomal recessive inheritance (18, 19). Even within the same gene, such as *RET*, different mutation types can result in distinct clinical outcomes: patients with the c.341G > A (p. Arg114His) mutation tend to have a benign disease phenotype, whereas the c.1852 T > C (p. Cys618Arg) mutation is more likely to be associated with a poor prognosis (20, 21).

### 3.4 CIPO is the manifestation of gastrointestinal involvement in certain hereditary syndromes

In some cases, CIPO is the manifestation of gastrointestinal involvement in certain hereditary syndromes. In our literature review, 46.67% (35/75) patients had syndromes, and hereditary CIPO just acts as the gastrointestinal manifestation among multi-system involvement. 44.83% (13/29) myogenic patients, 100% (17/17) patients with mitochondrial disorders, and 20.00% (5/25) neurogenic patients were reported with extra gastrointestinal symptoms. Megacystis-microcolon-intestinal Hypoperistalsis Syndrome (MMIHS), Mitochondrial Neurogastrointestinal Encephalopathy Disease (MNGIE), and Mitochondrial Encephalomyopathy with Lactic Acidosis and Stroke-like Episodes (MELAS) were three common phenotypic syndromes (Table 3). According to the data, 91.67% (11/12) of patients with MMIHS had *ACTG2* mutations, and MMIHS patients accounted for 52.38% (11/21) of all *ACTG2* mutation cases. Most MMIHS patients presented in the neonatal period (75%, 9/12), with urological symptoms such as megacystis, ureteral dilation, and hydronephrosis appearing early, and 91.67% patients (11/12) were detected by prenatal urological ultrasound. Among the 10 MNGIE patients with *TYMP* mutations in our study, all exhibited isolated small intestinal involvement and presented with neurological symptoms such as muscle weakness and sensory loss (22). In 4 MELAS patients caused by the *A3243G* mutation, CIPO appeared in the late clinical stage of the disease. These patients exhibited distinctive clinical features such as stroke-like episodes and hyperlactatemia.

**Table 3:** Phenotypic syndromes associated with hereditary CIPO patients.

Site of involvement	Number	Isolated small intestine involvement (*n* = 34)	Involvement of both the small and large intestines (*n* = 41)
Myogenic CIPO			
MMIHS	12	0	12 (29.27%)
EVC	1	0	1 (2.44%)
CIPO associated with mitochondrial disorders			
MNGIE	10	10 (29.41%)	0
MELAS	4	0	4 (9.76%)
MIDD	2	0	2 (4.88%)
HSAS	1	0	1 (2.44%)
Neurogenic CIPO			
CSBS	2	1 (2.94%)	1 (2.44%)
CCHS	1	0	1 (2.44%)
WS4	1	0	1 (2.44%)
PTHS	1	0	1 (2.44%)
Total	35	11	24

MMIHS, megacystis-microcolon-intestinal hypoperistalsis syndrome; EVC, Ellis-van Creveld syndrome; MNGIE, mitochondrial neurogastrointestinal encephalopathy disease; MELAS, mitochondrial encephalomyopathy with lactic acidosis and stroke-like episodes; MIDD, maternally inherited diabetes and deafness; HSAS, hydrocephalus with stenosis of the aqueduct of Sylvius; CSBS, congenital short bowel syndrome; CCHS, congenital central hypoventilation syndrome; WS4, Waardenburg syndrome IV; PTHS, Pitt-Hopkins syndrome.

## 4 Discussion

**Figure 3:**
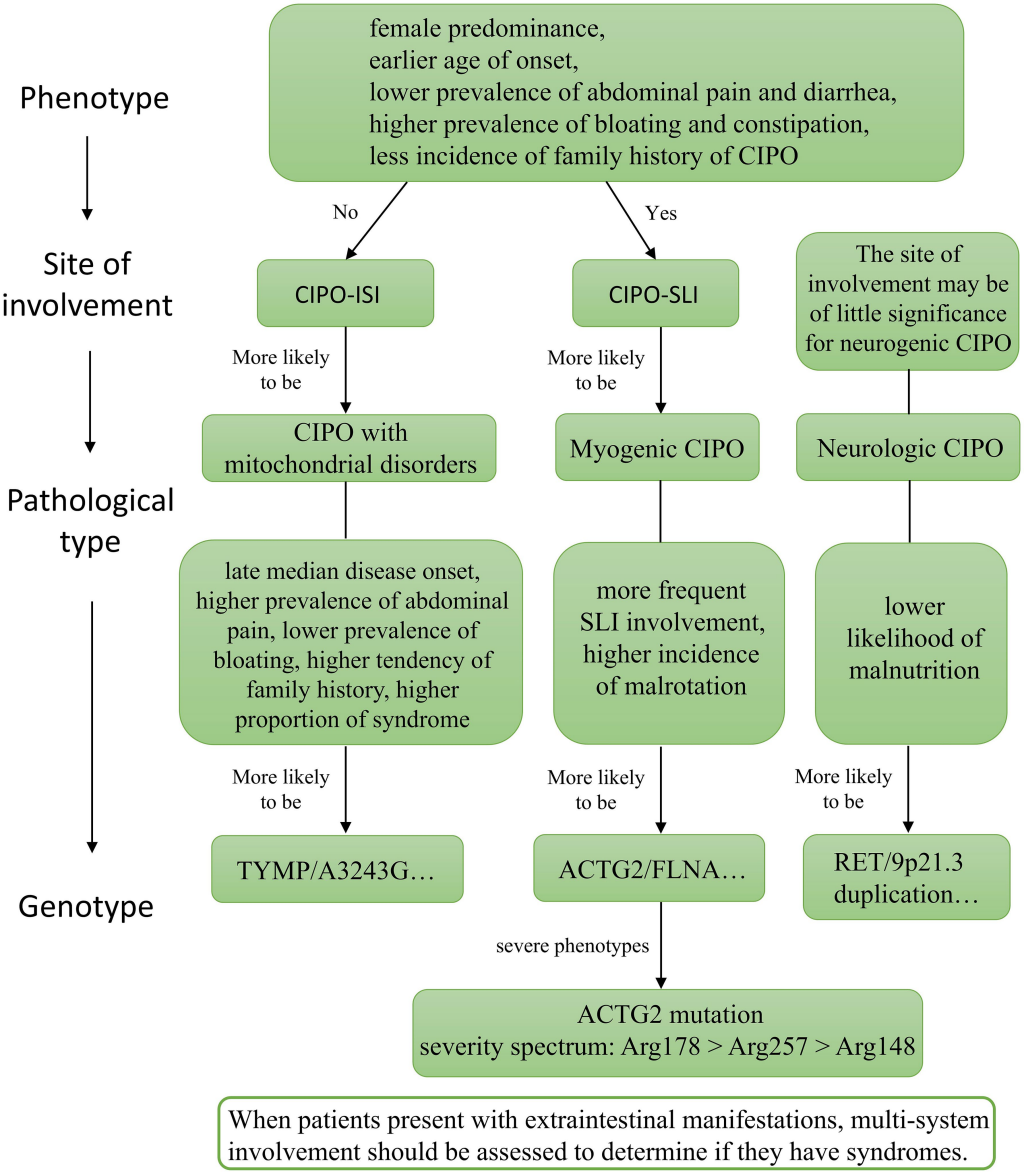
Flow chart showing possible phenotype and genotype correlations in hereditary CIPO patients with small intestine involvement.

This review systematically described the demographic, clinical, and genetic characteristics of 75 hereditary CIPO patients to uncover potential correlations between genotypes and phenotypes. Unlike previous studies that merely presented overall patient characteristics, this review categorizes patients into different groups according to affected regions and pathological types, conducting comparisons to explore the underlying regularity of phenotypic diversity (Figure 3). Firstly, compared with CIPO-ISI, CIPO-SLI had a female predominance, earlier age of onset, lower prevalence of abdominal pain and diarrhea, higher prevalence of bloating and constipation, and less incidence of family history of CIPO. Secondly, there were many differences among patients with different pathological types. Compared with myogenic CIPO, CIPO with mitochondrial disorders had late median disease onset, higher prevalence of abdominal pain, lower prevalence of bloating, and increased tendency of family history. Myogenic CIPO patients had more frequent SLI involvement and a high incidence of malrotation. Neurologic CIPO had diagnosis delay and less malnutrition. Finally, sometimes hereditary CIPO acts as the gastrointestinal manifestations of certain syndromes, including MMIHS, MNGIE, MELAS, etc. When diagnosing hereditary CIPO, these syndromes should be taken into account, and other systems should be evaluated.

In this article, we focus on hereditary CIPO with small intestine involvement due to its unique characteristics. Given the small intestine’s critical role in digestion and absorption and its high energy demands, mitochondrial energy metabolism disorders significantly impact small intestine motility, presenting as a special etiology for these patients (23). Additionally, the dysfunction of small intestine will definitely lead to severe clinical conditions, probably need for parenteral nutrition, and increase the risk of surgery (7). These characteristics collectively determine the need for clinical attention in hereditary CIPO patients with small intestine involvement.

Differences in symptoms can provide valuable clues to assess the site involved. The specificity of the physiological structure and function of different intestinal segments determines that their clinical manifestations will vary when lesions occur. The higher proportion of bloating and constipation in CIPO-SLI group might be related to the impaired motility function of large intestine and excessive water absorption of feces due to prolonged retention. Dilation, dysmotility, and fecal retention collectively cause significant bloating in these patients. Identifying the involved site in hereditary CIPO is not merely an anatomical issue but also a bridge linking etiology, phenotype, and treatment. In the study by Masaki et al. (7), the relationship between the involved site and the effectiveness of surgical treatment was revealed. Surgical interventions showed the highest efficacy in the large bowel group, moderate efficacy in the small bowel group, and the lowest efficacy in CIPO-SLI (7). Precise localization enables clinicians to achieve etiology-oriented diagnosis, individualized treatment, and prognosis stratification.

Based on the clinical presentation and information about the site involved, the pathological type is possible to be further speculated. For example, CIPO-ISI is more likely to be associated with mitochondrial disorders, while CIPO-SLI has a higher probability of myogenic type. According to previous studies, the *ACTG2*-positive group in myogenic CIPO patients always presented a severe clinical presentation and poor prognosis, which may be closely associated with arginine missense mutations (6). The severity spectrum of phenotypes caused by arginine substitutions is Arg178 > Arg257 > Arg148, with T149R and E196D related to the introduction of Arg residues also causing severe phenotypes (24, 25). While Matera et al. (26) reported that p. Arg257 is associated with CIPO and megacystis, our statistical analysis of *ACTG2* patients in this study failed to build a direct link between p. Arg257 and megacystis (Figure 2A). Additionally, patients with mitochondrial disorders exhibited a later age of onset, likely because mitochondrial dysfunction develops gradually. CIPO associated with mitochondrial disorders is caused by mtDNA depletion, a process involving point mutations, nucleotide pool imbalance, and mtDNA loss. Gastrointestinal motility disorders manifest only after sufficient accumulation of mtDNA defects. Furthermore, mitochondrial proliferation initially compensates for energy deficiencies caused by mtDNA depletion. When this compensation fails, smooth muscle cell function deteriorates, and gastrointestinal motility disorders gradually emerge (23). Prior studies have shown that p. V208M causes partial loss of thymidine phosphorylase activity, leading to late-onset disease, but this mutation was not included in our study (27, 28).

Notably, many hereditary CIPO patients present with multi-system symptoms, with gastrointestinal disease being just one manifestation (29). When hereditary CIPO is preliminarily identified based on clinical presentation, site of involvement, and pathology type, multi-system involvement should be assessed to determine if the patient has a syndrome. Through the discovery of syndromes and the analysis of extraintestinal manifestations, clinicians can better understand the pathophysiological mechanism and further support previous diagnoses. CIPO associated with mitochondrial disorders often exhibits more prominent multi-system involvement, a higher proportion of syndrome patients, and a poorer prognosis (30). Neurogenic CIPO is relatively less common. Moreover, certain syndromes can guide prognosis assessment. Heneyke et al. have reported that intestinal malrotation and urological involvement in MMIHS are poor prognostic factors for hereditary CIPO, showing important guiding significance for the screening of suspicious patients (31, 32, 73).

We re-evaluated the pathogenicity of Clinvar-classified VUS mutations according to the American College of Medical Genetics and Genomics and the Association (ACMG) guidelines. The p. Thr149Arg was reclassified from VUS to pathogenic, while the others remained largely unchanged (33).

While this review preliminarily explores the relationship between clinical phenotype and genotype of hereditary CIPO, several limitations warrant cautious interpretation. First, we only obtained a small sample size due to the rarity of hereditary CIPO cases and strict inclusion criteria. Additionally, the literature search was confined to English-language articles in PubMed, and incomplete information in some case series reports led to data missing. In account of the small sample size and the high heterogeneity of clinical symptoms, establishing a correlation between genotype and phenotype is still challenging. We need to emphasize the exploratory nature of this review, and its conclusions still require further verification through additional research and case accumulation. Second, pediatric and adult cases were not analyzed separately, limited by the small case number. Third, in the established relationships between heredity and phenotype, complex phenotypes are often oversimplified. For example, different *RET* mutation sites leading to varying prognoses may not only be caused by CIPO but also by the involvement of extraintestinal systems. It is necessary to systematically evaluate the cumulative effects of multi-organ involvement on prognosis and identify the core systems that play a crucial role. Lastly, not all variations recorded are pathogenic or likely pathogenic. There are also some benign, non-sensical, or yet to be included in Clinvar. Further exploration and evaluation of these mutations are needed.

In summary, this article collected and analyzed reported cases of hereditary CIPO with small intestine involvement over the past 25 years, elucidating the disparity of genotype and phenotype among patients. It is hoped that these findings will offer valuable insights for the early identification of hereditary CIPO, thereby reducing surgical interventions resulting from misdiagnosis or unclear diagnoses.
