# Supplementary material for: Phenotype and genotype in hereditary chronic intestinal pseudo-obstruction with small intestine involvement
Source: Front Med (Lausanne). 2025 Aug 21;12:1632816. doi: 10.3389/fmed.2025.1632816 (PMC12408701; doi:10.3389/fmed.2025.1632816)
Supplement: Supplementary file 1 [file Table_1.docx]

**Supplementary material**

Search query in PubMed:

(((((small[Title/Abstract] OR total[Title/Abstract]) AND (intestine*[Title/Abstract] OR bowel*[Title/Abstract] OR gut[Title/Abstract] OR enter*[Title/Abstract])) OR Duoden*[Title/Abstract] OR ileu*[Title/Abstract] OR ileo*[Title/Abstract] OR jejun*[Title/Abstract]) AND ("Hirschsprung's disease"[Title/Abstract] OR aganglionosis[Title/Abstract] OR hypoganglionosis[Title/Abstract])) OR "Intestinal Pseudo Obstruction*"[Title/Abstract] OR "Intestinal Pseudo-Obstruction*"[Title/Abstract] OR "Intestinal Pseudoobstruction*"[Title/Abstract] OR "Pseudoobstruction, Intestinal"[Title/Abstract] OR "Pseudo-Obstruction, Intestinal"[Title/Abstract] OR "Pseudo Obstruction, Intestinal"[Title/Abstract] OR "Pseudoobstructive Syndrome*"[Title/Abstract] OR "Idiopathic Intestinal Pseudo-Obstruction*"[Title/Abstract] OR "Paralytic Ileus"[Title/Abstract] OR "Visceral Myopath*"[Title/Abstract] OR "Congenital Short Bowel Syndrome"[Title/Abstract] OR "Enteric Neuropath*"[Title/Abstract] OR "Chronic Idiopathic Intestinal Pseudo-Obstruction"[Title/Abstract] OR "Chronic Idiopathic Intestinal Pseudo Obstruction"[Title/Abstract] OR "CIPO"[Title/Abstract] OR "Intestinal Pseudo-Obstruction"[Mesh]) AND (gene*[Title/Abstract] OR heredit*[Title/Abstract] OR mutation*[Title/Abstract] OR variant*[Title/Abstract] OR chromosome*[Title/Abstract] OR "Mutation"[Mesh] OR "Chromosomes"[Mesh] OR "Genes"[Mesh] OR "Genetics"[Mesh]) AND (english[Filter]) AND (human* OR patient* OR individual* OR man OR men OR woman OR women OR girl* OR boy OR child* OR kid OR infant* OR newborn* OR neonat*)
